# Intensive antithrombotic therapy is necessary for long-term treatment in patients with symptomatic peripheral artery disease after acute myocardial infarction

**DOI:** 10.1038/s41598-025-24372-2

**Published:** 2025-11-07

**Authors:** Jan Matejka, Zuzana Motovska, Ota Hlinomaz, Petr Kala, Milan Hromadka, Ivo Varvarovsky, Jaroslav Dusek, Jiri Jarkovsky, Richard Rokyta, Jan Mrozek, Pavel Cervinka, Stanislav Simek, Jiri Ostransky

**Affiliations:** 1 Dept. of Cardiology, Pardubice Hospital, Pardubice, Czech Republic; 2 Cardiocenter, Third Faculty of Medicine, Charles Univ. and Univ. Hospital Kralovske Vinohrady, Srobarova 50, 100 34 Prague, Czech Republic; 3 First Dept. of Intern. Medicine – Cardioangiology, Faculty of Medicine, Masaryk University and St. Anne’s University Hospital, Brno, Czech Republic; 4 Dept. of Internal Medicine and Cardiology, Faculty of Medicine of Masaryk University and Univ. Hospital Brno, Brno, Czech Republic; 5 Dept. of Cardiology, Univ. Hospital and Faculty of Medicine in Pilsen, Charles University, Prague, Czech Republic; 6 Cardiology Center AGEL, Pardubice, Czech Republic; 7 Dept. of Cardiovascular Medicine I, University Hospital Hradec Kralove, Hradec Kralove, Czech Republic; 8 Institute of Biostatistics and Analysis, Faculty of Medicine, Masaryk University, Brno, Czech Republic; 9 Cardiovascular Department, University Hospital, Ostrava, Czech Republic; 10 Second Dept. of Internal Medicine, First Faculty of Medicine, General Teaching Hospital, Charles University, Cardiovascular Medicine, Prague, Czech Republic; 11 Dept. of Internal Medicine I - Cardiology, Faculty of Medicine and Dentistry, Palacky University and Univ. Hospital, Olomouc, Czech Republic

**Keywords:** Acute myocardial infarction, Peripheral artery disease, Antiplatelet therapy, Ticagrelor, Prasugrel, Cardiology, Myocardial infarction

## Abstract

Patients with acute myocardial infarction (AMI) who have concomitant peripheral artery disease (PAD) represent a subgroup at high risk of subsequent ischaemic events. This post hoc analysis of PRAGUE-18, a multicenter, randomised trial comparing prasugrel versus ticagrelor in primary PCI, analysed the effect of symptomatic PAD and intensity of antithrombotic therapy on the prognosis of AMI patients treated with primary percutaneous coronary intervention (PCI). During 12-month follow-up, de-escalation from intensive antiplatelet therapy to clopidogrel was allowed with the permission of the treating physician for economic reasons. Symptomatic PAD was present in 2.9% of the study population (n = 1230). The presence of PAD did not significantly affect short-term outcome. At one year, the risk of death was higher in patients with concomitant PAD, 49 (4.1%) vs. 6(16.7%), HR 4.211 (1.803–9.830); p = 0,001. All-cause mortality significantly increased only in subgroup of patients who de-escalated to clopidogrel [6.37 (2.16–18.84), p = 0.001] as opposed to those who did not [3.02 (0.72–12.61), p = 0.13]. These findings suggest that long-term intensive antithrombotic therapy may be particularly important for post-AMI patients with concomitant symptomatic PAD and warrant further investigation.

## Introduction

Acute ST-elevation myocardial infarction (STEMI) represents a clinical entity with a very high thrombotic risk^1^. Mortality in patients with STEMI is influenced by many factors including age, Killip classification, time to treatment delay, prehospital care organisation (emergency medical services-based STEMI networks), treatment strategy, history of myocardial infarction, diabetes, renal impairment, degree of coronary artery involvement, and left ventricular ejection fraction^2^. Despite numerous recent improvements, mortality remains significant. Hospital mortality varies between 4 and 12%, and reported 1-year mortality is 9.6 -13.3%^3^.

The most effective treatment for STEMI, when available, is primary percutaneous coronary intervention (PCI) with stent implantation leading to a restoration of antegrade flow in the infarct-related artery^4^. Adjuvant dual therapy with acetylsalicylic acid combined with the potent P2Y12-platelet receptor inhibitor, ticagrelor or prasugrel, for 12 months is recommended by international guidelines in the absence of contraindications or high bleeding risk^4–6^. De-escalation of dual therapy, i.e. switching from ticagrelor/prasugrel to clopidogrel, is considered because of the risk of bleeding, other adverse effects (dyspnoea), and socioeconomic reasons. It may be considered in patients at high risk of bleeding over 30 days after an acute coronary syndrome^4^.

A characteristic feature of atherothrombotic diseases is the co-prevalence of atherosclerotic involvement of multiple arterial beds – polyvascular involvement^7^. Peripheral artery disease (PAD), as another manifestation of atherothrombotic disease, shares major risk factors with coronary artery disease (CAD)^8^. Patients with PAD have concomitant CAD in 50% of cases, and patients with CAD have concomitant PAD in 20% of cases^9^. PAD is associated with increased subsequent coronary events, cardiovascular and overall mortality even after adjustment for known common risk factors^10^. The presence of PAD also leads to worse long-term outcomes after PCI^11^. In patients with symptomatic PAD, antiplatelet therapy improves cardiovascular prognosis^12^. The assumption that the coexistence of PAD in patients with AMI further increases ischaemic risk leads us to use more effective antithrombotic regimens in terms of duration and intensity of treatment^13^. In the PEGASUS-TIMI 54 (Prevention of Cardiovascular Events in Patients with Prior Heart Attack Using Ticagrelor Compared to Placebo on a Background of Aspirin-Thrombolysis in Myocardial Infarction 54) trial investigating the addition of ticagrelor to aspirin in patients after myocardial infarction in the previous 1–3 years, 5% of patients had PAD. In this subgroup, the largest (4.1%) absolute decrease in major adverse cardiovascular events (MACE) was observed^13^. In addition to prolonging antithrombotic therapy, intensifying antithrombotic therapy is therefore considered in these patients. In the CAPRIE (a randomised, blinded trial of clopidogrel versus aspirin in patients at risk of ischaemic events) trial, monotherapy with clopidogrel was more effective compared to aspirin in preventing MACE in the PAD subgroup of patients after MI^14^.

Post hoc analyses of PAD patients enrolled in the PLATO (The Study of Platelet Inhibition and Patient Outcomes) and COMPASS (Cardiovascular Outcomes for People Using Anticoagulation Strategies) trials showed a reduction in ischaemic endpoints consistent with the overall results of these studies^15,16^.

As mentioned above, patients with STEMI and concomitant PAD represent a subgroup at the highest risk of subsequent ischaemic events. However, the evidence for supporting intensive combined antithrombotic therapy in this subgroup of patients is ambiguous^13,17^. Furthermore, most of the published work has focused on the co-occurrence of PAD and chronic coronary syndromes^9^.

The aim of our study was to analyse the effect of symptomatic PAD on the prognosis of patients with AMI treated with primary PCI and intensive antiplatelet therapy.

## Methods

A post hoc analysis of patients enrolled in the Prague-18 study was performed. In brief, the Prague-18 study was a non-commercial academic multicenter randomised open-label trial comparing the efficacy and safety of ticagrelor and prasugrel in patients treated with primary percutaneous coronary intervention (PCI) for AMI. A total of 1230 patients were enrolled in the study. The study was approved by the Committee for Multicenter Clinical Trials, University Hospital Kralovske Vinohrady, Prague, Czech Republic and the corresponding local committees at each study center. The study protocol was registered as PRAGUE-18 (http://www.ClinicalTrials.gov; NCT02808767). Informed consent was obtained from all participants. Research has been performed in accordance with the Declaration of Helsinki. The design and results of the study are described in detail in previous publications^18,19^.

Study patients. Patients with acute myocardial infarction treated with primary PCI for acute STEMI and high-risk non-ST-elevation acute MI (NSTEMI) were included in the study. Inclusion criteria were urgent coronary angiography within 120 min of admission to the cardiac center and the provision of informed consent. Exclusion criteria for the study were history of stroke, serious bleeding within the past 6 months, indication for long-term oral anticoagulation therapy, administration of clopidogrel ≥ 300 mg or any other antiplatelet medication (except aspirin and a lower dose of clopidogrel) before randomisation, age > 75 years and body weight < 60 kg, moderate or severe hepatic function disorder, concomitant treatment with a strong CYP3A4 inhibitor, and known hypersensitivity to prasugrel or ticagrelor. Patients were divided into two groups for post-hoc analysis according to the presence or absence of symptomatic PAD. PAD was defined as intermittent claudication, chronic critical limb ischaemia, acute limb ischaemia, or previous lower limb revascularisation, as confirmed by the treating cardiologist or angiologist in the medical records.

Treatment and follow-up. Patients received a loading dose of ticagrelor or prasugrel immediately after admission to hospital (usually in the catheterisation laboratory) and were advised to continue this treatment for 12 months in combination with 100 mg of aspirin daily. Both the loading dose and maintenance doses of the drugs followed current recommendations^4^. Whether patients completed intensive antiplatelet therapy throughout the 1-year follow-up period or de-escalated from the therapy for economic or non-economic reasons was analysed. The study protocol allowed patients, with the consent of the treating physician, to de-escalate from the long-term study medication to clopidogrel for economic reasons. Unlike ticagrelor or prasugrel, clopidogrel is fully reimbursed by public health insurance. Non-economic causes of de-escalation included the need for chronic oral anticoagulation therapy, adverse drug reactions, or other reasons. Patient visits were determined as follows: initial contact at the time of randomisation, visits on day 7 of hospitalisation or at discharge if before day 7, day 30 (telephone visit), and 1 year from the index event.

The primary endpoint was the occurrence of death, reinfarction, urgent target vessel revascularisation, stroke, severe bleeding requiring transfusion, or prolonged hospitalisation. The following endpoints were monitored: combined ischaemic endpoint (cardiovascular death, MI, stroke), individual components of the combined ischaemic endpoint, all-cause death, and bleeding. Bleeding was defined according to TIMI (Thrombolysis in Myocardial Infarction) and BARC (Bleeding Academic Research Consortium) criteria^20^.

Statistical analysis. Standard descriptive statistics were applied in the analysis, absolute and relative frequencies for categorical variables and median supplemented with 5-95^th^ percentile for continuous variables. The statistical significance of differences between groups of patients was tested using the Fisher exact test for categorical variables, and the Mann–Whitney U test for continuous variables. Time-to-event analysis was based on Cox proportional hazards regression model and described using hazard ratios supplemented with their 95% confidence intervals and statistical significance. All analyses were computed with a = 0.05 as a level of statistical significance, and SPSS 26.0.0.0 (IBM Corporation 2020) was used for the analysis.

## Results

A total of 1230 patients were included in the study. Symptomatic PAD was present in 36 (2.9%) of them. Baseline and procedural characteristics of patients are shown in Table 1**.** Compared with the group without PAD, patients with PAD had a higher prevalence of dyslipidaemia, history of myocardial infarction, previous PCI, previous coronary bypass surgery, chronic heart failure, left main coronary artery involvement, and lower left ventricular ejection fraction, and were also more likely to be taking beta-blockers, aspirin, and statins before randomisation.

**Table 1:** Baseline and procedural characteristics of study patients.

	**No PAD**	**PAD**	**P value**	**RR (95% CI)**
	N = 1194	N = 36		
**Acute coronary syndrome type**				
ST elevation	1 104 (92.5%)	31 (86.1%)	0.192	0.931 (0.816; 1.063)
Left bundle branch block	17 (1.4%)	1 (2.8%)	0.416	1.951 (0.267; 14.264)
Right bundle branch block	21 (1.8%)	2 (5.6%)	0.144	3.159 (0.770; 12.964)
No ST elevation	64 (5.4%)	2 (5.6%)	0.999	1.036 (0.264; 4.070)
**Baseline characteristics**				
Men	902 (75.5%)	29 (80.6%)	0.560	1.066 (0.905; 1.256)
Age	61.8 (43.4; 79.0)	64.3 (50.5; 73.6)	0.295	
BMI	27.8 (22.3; 36.3)	27.1 (22.1; 33.9)	0.200	
Killip class 1	1 055 (88.4%)	30 (83.3%)	0.163	0.943 (0.814; 1.093)
2	79 (6.6%)	3 (8.3%)		1.259 (0.417; 3.800)
3	15 (1.3%)	2 (5.6%)		4.422 (1.050; 18.622)
4	45 (3.8%)	1 (2.8%)		0.737 (0.104; 5.200)
**Risk factors and comorbidies**				
Dyslipideamia	403 (33.8%)	19 (52.8%)	**0.021**	1.564 (1.137; 2.151)
Obesity	231 (19.3%)	9 (25.0%)	0.395	1.292 (0.725; 2.302)
Hypertension	606 (50.8%)	24 (66.7%)	0.064	1.314 (1.036; 1.666)
Smoking	770 (64.5%)	28 (77.8%)	0.112	1.206 (1.008; 1.443)
Diabetes	239 (20.0%)	11 (30.6%)	0.140	1.526 (0.921; 2.530)
History of myocardial infarction	88 (7.4%)	15 (41.7%)	** < 0.001**	5.653 (3.657; 8.740)
History of PCI	76 (6.4%)	11 (30.6%)	** < 0.001**	4.800 (2.802; 8.224)
History of CABG	18 (1.5%)	3 (8.3%)	**0.021**	5.528 (1.705; 17.926)
Chronic heart failure	10 (0.8%)	2 (5.6%)	**0.046**	6.633 (1.508; 29.185)
Chronic kidney disease	15 (1.3%)	1 (2.8%)	0.380	2.211 (0.300; 16.288)
Bleeding	6 (0.5%)	0 (0.0%)	0.999	
**Medication before randomization**				
Aspirin	166 (13.9%)	26 (72.2%)	** < 0.001**	5.195 (4.058; 6.650)
Betablockers	213 (17.8%)	14 (38.9%)	**0.003**	2.180 (1.422; 3.342)
Angiotensin-coverting-enzyme inhibitors	271 (22.7%)	11 (30.6%)	0.313	1.346 (0.814; 2.227)
Angiotensin-receptor blockers	127 (10.6%)	5 (13.9%)	0.580	1.306 (0.569; 2.994)
Statins	202 (16.9%)	16 (44.4%)	** < 0.001**	2.627 (1.785; 3.866)
Protone pump inhibitors	71 (5.9%)	5 (13.9%)	0.066	2.336 (1.004; 5.433)
**Coronary angiography and PCI**				
Infarct-related artery TIMI flow after PCI < 3	56 (4.7%)	1 (2.8%)	0.999	0.592 (0.084; 4.160)
Number of diseased vessels > 1	603 (50.6%)	21 (58.3%)	0.400	1.155 (0.871; 1.531)
Left main disease	38 (3.2%)	3 (8.3%)	0.115	2.618 (0.848; 8.087)
Infarct-related artery				
Left main	10 (0.8%)	2 (5.6%)	**0.046**	6.633 (1.508; 29.185)
Left anterior descending	460 (38.5%)	16 (44.4%)	0.491	1.154 (0.795; 1.674)
Left anterior descending/diagnonal	67 (5.6%)	2 (5.6%)	0.999	0.990 (0.252; 3.884)
Circumflex	133 (11.1%)	2 (5.6%)	0.419	0.499 (0.128; 1.936)
Circumflex/marginal	85 (7.1%)	2 (5.6%)	0.999	0.780 (0.200; 3.048)
Right coronary artery	495 (41.5%)	18 (50.0%)	0.309	1.206 (0.864; 1.684)
**Echocardiography**				
LVEF	50.0 (30.0; 65.0)	40.0 (25.0; 60.0)	**0.001**	

**PAD** – peripheral artery disease; **RR (95% CI)** – relative risk (95% confidence interval) PAD vs. no PAD; **BMI** – body mass index ; **PCI** – percutaneous coronary intervention; **CABG** – coronary artery bypass graft; **LVEF** – left ventricular ejection fraction.

Categorical parameters are represented as absolute (relative) frequencies and tested with Fisher exact test.

Continuous parameters are represented as median (5th;95th percentile) and tested with Mann–Whitney test.

Background prognostic medication was adequately initiated at hospital discharge, with more than 80% taking beta-blockers, renin-angiotensin receptor inhibitors and statins. Analysis of medication prescriptions up to 30 days after study enrollment showed that the groups with and without PAD did not differ in the representation of betablockers, 902 (77.3%) vs. 25 (69.4%); p = 0.313, and renin-angiotensin receptor inhibitors, 873 (74.8%) vs. 24 (66.7%); p = 0.330. Prescription of statins was slightly lower in the group of patients with PAD, 1013 (86,8%) vs. 25 (69,4%); p = 0,006.

De-escalation of treatment occurred in a total of 659 (53.6%) patients during follow-up: for economic reasons in 481 (39.1%) patients and the remaining 178 (14.5%) for non-economic reasons. The percentage of patients who de-escalated intensive antiplatelet treatment for economic reasons was similar among those without PAD and with PAD 466 (39.0%) vs. 15 (41.7%); p = 0.733.

**Fig. 1:**
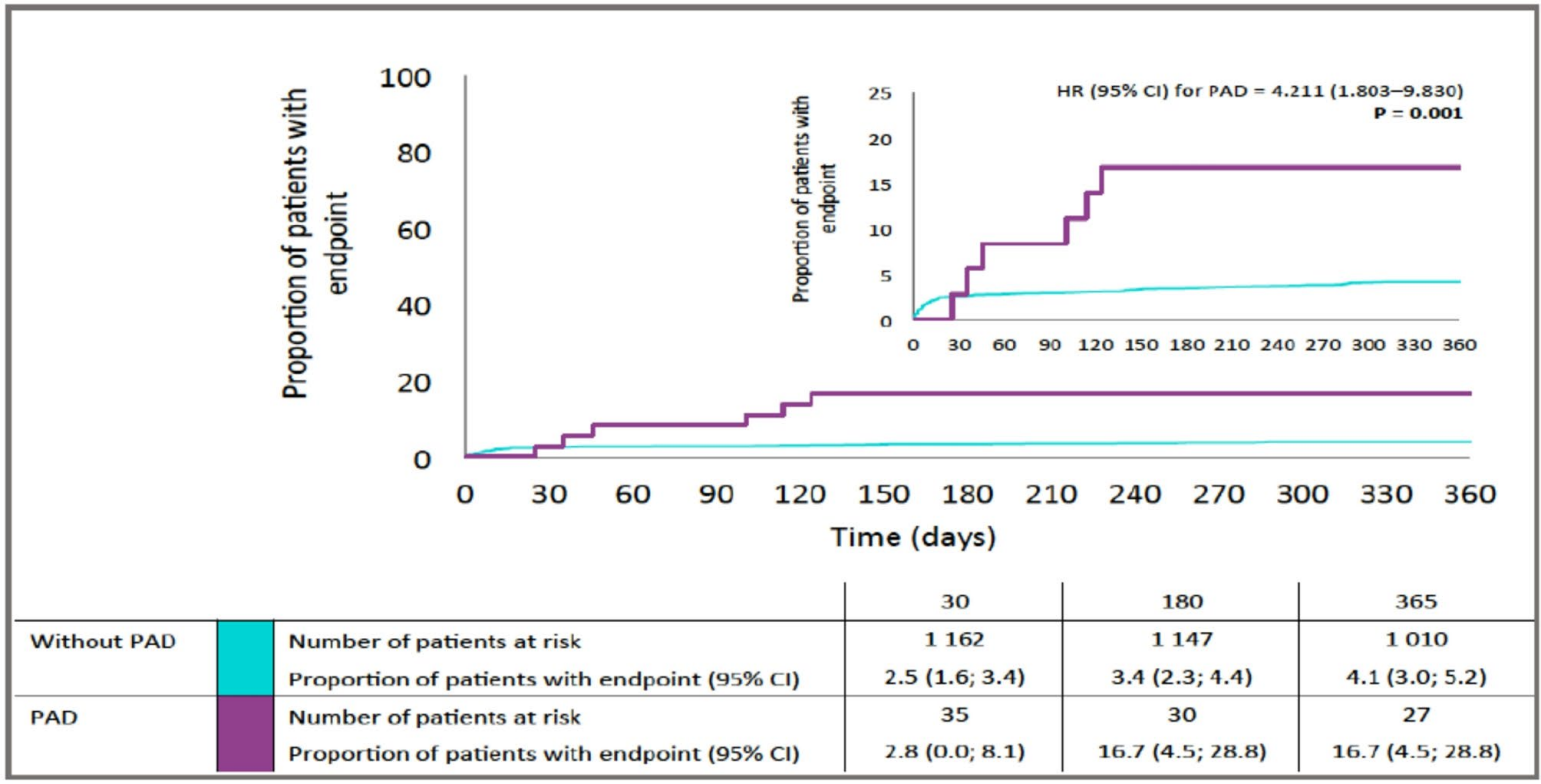
Mortality in Relation to the Presence of Peripheral Artery Disease. PAD—peripheral artery disease; End Point—all-cause mortality.

In the early post-randomisation period, no significant difference related to the presence of PAD was observed in either the primary endpoint at seven days or in the combined ischaemic endpoint, cardiovascular death, and all-cause death at 30 days (Table 2). Looking at the long-term results at one year, the risk of death was higher in patients with concomitant PAD **(**Fig. 1**, **Table 2**)**.

**Table 2:** Study end points.

Endpoint	No PAD	PAD	HR (95% CI)	P value
	**N = 1194**	**N = 36**		
**Day 7**				
Primary end point	48 (4.1%)	1 (2.8%)	0.704 (0.097–5.100)	0.728
**Day 30**				
Combined ischemic end point	39 (3.3%)	2 (5.6%)	1.705 (0.412–7.061)	0.462
Cardiovascular death	24 (2.0%)	1 (2.8%)	1.368 (0.185–10.109)	0.759
All-cause death	30 (2.5%)	1 (2.8%)	1.093 (0.149–8.011)	0.931
**Day 365**				
Combined ischemic endpoint	72 (6.0%)	4 (11.1%)	1,929 (0.705–5.280)	0.201
Cardiovascular death	36 (3.0%)	3 (8.3%)	2,845 (0.876–9.240)	0.082
All-cause death	49 (4.1%)	6 (16.7%)	4.211 (1.803- 9.830)	**0.001**
Bleeding	132 (11.1%)	3 (8.3%)	0.775 (0.247–2.433)	0.662

**PAD –** peripheral artery disease **HR –** hazard ratio (95% confidence interval)** ; Primary end point—**death, reinfarction, urgent target vessel revascularization, stroke, severe bleeding requiring transfusion or prolonged hospitalization; **Combined ischemic end point**—death, myocardial infarction or stroke.

Categorical parameters are represented as absolute (relative) frequencies and tested with Fisher exact test. The hazard ratio was based on the Cox proportionals hazard model.

However, the occurrence of this endpoint was influenced by the de-escalation of potent antiplatelet treatment. The risk of death was significantly increased only in patients who de-escalated to clopidogrel [6.37 (2.16–18.84), p = 0.001]. The difference in risk of death in patients with and without PAD who continued ticagrelor/prasugrel did not reach significance [3.02 (0.72–12.61), p = 0.13] **(**Table 3**)**. The landmark analysis showed that from day 30 up until the end of the study, the risks of cardiovascular death and all-cause death were significantly higher in PAD patients [6.13 (1.37–27.38), p = 0.018 and 9.66 (3.61–25.87), p < 0.001 respectively]. As in the previous case, the significant impact of PAD on the long-term outcome was present only in patients who switched to clopidogrel. No difference was found in the occurrence of bleeding events between patients with (8.3%) and without (11.1%) PAD **(**Table 2**)**.

**Table 3:** Study end points in relation to de-escalation of antiplatelet therapy.

	No PAD	PAD	HR	P value
	N = 1194	N = 36		
**No treatment de-escalation**	559 (46.8%)	12 (33.3%)	–	0.128
All cause death day 30	25 (4.5%)	1 (8.3%)00	1.83 (0.248–13.483)	0.555
Combined ischemic end point day 365	41 (7.3%)	2 (16.7%)	2.314 (0.560–9.567)	0.247
Cardiovascular death day 365	25 (4.5%)	2 (16.7%)	3.731 (0.884–15,752)	0.073
All cause death day 365	31 (5.5%)	2 (16.7%)	3.02 (0.722–12.614)	0.130
**Treatment de-escalation**	635 (53.2%)	24 (66.7%)	–	0.128
Time to treatment de-escalation (days)	11.0 (2.0; 226.0)	7.5 (2.0; 214.0)	–	0.448
Treatment de-escalation due to economic reasons within 335 days	466 (39.0%)	15 (41.7%)	–	0.733
All cause death day 30	5 (0.8%)	0 (0.0%)	–	–
Combined ischemic end point day 365	31 (4.9%)	2 (8.3%)	1.831 (0.438–7.651)	0.407
Cardiovascular death day 365	11 (1.7%)	1 (4.2%)	2.638 (0.340–20.442)	0.353
All cause death day 365	18 (2.8%)	4 (16.7%)	6.37 (2.156–18.838)	**0.001**

**PAD**—peripheral artery disease; **No treatment de-escalation**—patients who remained on potent antiplatelet therapy (ASA + ticagrelor/prasugrel) during the whole study duration; **Treatment de-escalation**—patients who switched from potent antiplatelet therapy (ASA + ticagrelor/prasugrel) to clopidogrel before study termination; **HR**—hazard ratio; **Combined ischemic end point**—cardiovascular death, myocardial infarction or stroke.

Categorical parameters are represented as absolute (relative) frequencies and tested with Fisher exact test. Continuous parameters are represented as median (5th;95th percentile) and tested with Mann–Whitney test. The hazard ratio was based on the Cox proportional hazard model.

## Discussion

A post hoc analysis of the PRAGUE-18 randomised trial, which included patients with AMI treated with primary PCI, showed that the presence of symptomatic PAD significantly affects the prognosis of patients after STEMI.

The proportion of patients with symptomatic PAD in the PRAGUE-18 trial enrolling patients after primary PCI was 2.9%. The prevalence of PAD is influenced by the definition used in different studies. It is higher if it includes asymptomatic patients with an abnormal ankle-brachial index in addition to symptomatic ones^15,21^. In our analysis, we considered the most severe, symptomatic forms of PAD. Therefore, the results of the presented analysis on the long-term benefit of intensive combined antiplatelet therapy apply only to patients with symptomatic PAD.

Patients with PAD in our study had a fourfold higher 1-year risk of death compared with those without PAD. This difference is more profound than in similar studies and may be attributed to the higher thrombotic risk of our cohort, which almost exclusively included patients with STEMI^9,11,22^.

The risk of PAD patients can be viewed in two ways. The first is from the perspective of morbidity directly associated with lower limb ischaemia. The second, which is the most relevant to our work, is in terms of systemic severe atherosclerotic complications related to the extent of atherosclerosis, the involvement of multiple arterial beds, but also to underdiagnosis and underuse of evidence-based treatments, including antiplatelet drugs, statins, and smoking cessation efforts^8,23^. The vast majority of all patients had optimal medical therapy for CAD secondary prevention at discharge^18^. There was no significant difference in the use of aspirin, beta-blockers, and ACE inhibitors in patients with or without PAD, nor a difference in the prescription rate of these medications after discharge, with the exception of statins, where slightly lower prescription at day 30 may indicate poorer treatment adherence in the PAD group. Thus, underuse of the best medical therapy in PAD patients was not observed in our study. The aforementioned influence of severe systemic atherosclerosis on the one hand and the intensity of antiplatelet therapy during the 12-month follow-up on the other hand played a more important role.

In the first month after randomisation, the incidence of the combined ischaemic endpoint or its individual components was not significantly higher in the group of patients with PAD compared with those without PAD. The following factors may have been involved. During this period, all participants were receiving intensive antithrombotic therapy, and de-escalation to clopidogrel occurred on average after more than seven days (Table 3). Moreover, patients with PAD had higher rates of statin, aspirin, and beta-blocker use at baseline.

Thus, we observed an increased risk of death of PAD patients in the later period (1–12 months after AMI). First and foremost, we emphasise the higher relative risk of death in patients with peripheral artery disease (PAD) compared to patients without PAD. The difference in overall mortality between patients with and without peripheral artery disease (PAD) was most pronounced and statistically significant among those who de-escalated potent anti-thrombotic therapy during the study. Considerations for de-escalation of potent antiplatelet therapy are not uncommon in today’s daily practice^4,23–25^. In addition to the consequences of bleeding complications or other adverse effects of the medication administered, concerns about bleeding risk or optimal PCI outcome with the assumption of low additional ischaemic risk may lead cardiologists to de-escalate therapy. On the patient side, economic considerations also play a substantial role. The original PRAGUE-18 study presented an algorithm for personalised care, a safe and controlled switch of antithrombotic therapy with ticagrelor or prasugrel in the maintenance phase of AMI treatment to clopidogrel. The selection of low-risk patients was an essential condition for de-escalation of treatment^18^. Our sub-analysis suggests that de-escalating therapy in high-risk patients with AMI and concomitant symptomatic PAD may negatively impact patient outcomes.

Our analysis has several limitations. The number of patients in the PAD group is small, and their division into subgroups of intensive treatment and de-escalation makes statistical evaluation difficult. The findings are merely hypothesis-generating and may form a basis for further research. Nevertheless, the results demonstrate a clinically significant trend consistent with previous studies (PEGASUS-TIMI 54, PLATO and COMPASS).

However, the highest-risk PAD (symptomatic PAD) subjects were included in the analysis. The results depict a well-defined population, with controlled treatment and accurately defined risks of events for patients with and without symptomatic peripheral artery disease (PAD). All the endpoints were adjudicated by an event adjudication committee and the data originate from a monitored and carefully conducted previously published randomised trial with a clearly defined population. Treatment de-escalation from potent antiplatelet drugs to clopidogrel was not a randomised process. Treatment de-escalation was decided by patients and influenced by economic (reimbursement) factors, thus reflecting everyday practice.

## Conclusion

A post-hoc analysis of the PRAGUE-18 trial demonstrated that the presence of symptomatic PAD significantly affects the prognosis in patients treated with primary PCI for acute myocardial infarction. The difference in outcomes between patients with and without PAD was most evident among those who de-escalated potent anti-thrombotic therapy. These findings suggest that long-term intensive antithrombotic therapy may be particularly important for post-AMI patients with concomitant symptomatic PAD and provide a foundation for future research in this area.

## Data Availability

The datasets generated during and/or analysed during the current study are not publicly available due to individual data privacy protection but are available from the corresponding author on reasonable request.
